# 分子诊断技术在静脉血栓栓塞症中的研究进展

**DOI:** 10.3760/cma.j.issn.0253-2727.2022.11.015

**Published:** 2022-11

**Authors:** 耀华 蔡, 君 邓, 豫 胡

**Affiliations:** 华中科技大学同济医学院附属协和医院血液科，武汉 430022 Department of Hematology, Union Hospital, Tongji Medical College, Huazhong University of Science and Technology, Wuhan 430022, China

静脉血栓栓塞症（VTE）包括深静脉血栓形成（DVT）和肺栓塞（PE），临床表现多样（如脑静脉窦VTE、肠系膜VTE）。流行病学调查结果示VTE发病率为每千人1～1.5，人均发生风险为11％，且VTE发生后5年复发率高达20％。VTE是由遗传、环境、生活习惯等多因素共同作用的疾病。研究表明遗传因素的贡献约为60％[1]。VTE与多种基因有关，累及全身多部位或脏器静脉系统，涉及多个临床学科，常起病隐匿，已成为仅次于心肌梗死及卒中的世界第三大循环系统致死性疾病。因此积极开展VTE遗传分析研究对于提高临床诊疗水平有重要意义[2]。

传统的VTE实验室检查主要依赖于检测表型，即生化指标的检测，但该类指标结果常常受不同抗凝药物和血栓病程的影响[3]。遗传变异因素是参与VTE发生、发展的关键要素，且不受药物及生理病理状态的影响。基因的分子诊断技术可以较为全面、准确地筛查VTE患者高危遗传因素，早期识别高危人群，指导临床用药和抗凝疗程的选择，临床应用前景光明。

分子诊断技术是指以脱氧核糖核酸（DNA）和核糖核酸（RNA）为诊断材料，通过分子生物学技术检测基因的存在、缺陷或表达异常，从而对人体状态和疾病作出诊断的技术。VTE相关的分子诊断技术主要包括PCR、基因芯片以及二代测序（NGS）等。

一、以PCR为基础的分子诊断技术

PCR是一种扩增特定DNA片段的分子生物学技术，通过体外模拟DNA合成从而在短时间内指数级扩增目标DNA。在此基础上派生出了众多新的用途，由PCR衍生出来的实时荧光定量PCR[4]–[5]、限制性片段长度多态性PCR[6]、扩增阻滞突变系统PCR[7]等技术也在VTE的基础研究中屡见报道。作为现阶段接受程度最高的临床分子检测技术，PCR在拷贝数变异、突变检测、核酸分子定量等方面都表现出快速和精确的优势。

二、以分子杂交为基础的分子诊断技术

（一）多重连接探针扩增技术（MLPA）

MLPA是一种探针依赖扩增技术，基本原理是将探针与靶序列DNA杂交，再进行PCR扩增，通过毛细管电泳对PCR产物进行鉴定，可以同时检测50多个不同靶基因。目前在基因拷贝数分析、单核苷酸多态性等（SNP）领域已被广泛应用[8]。

两项研究先后通过MLPA技术新发现了导致抗凝血酶缺乏症的5、8个SERPINC1突变[9]–[10]。Togashi等[11]对蛋白C缺乏症患者进行直接测序并未发现突变，然而MLPA的遗传分析表明患者PROC的第7、8个外显子缺失。MLPA技术具有高效性、特异性，可以发现常规检测技术所遗漏的基因位点。

（二）基因芯片技术

基因芯片又称DNA芯片或基因微阵列，随着人类基因组计划而逐渐发展成熟，其基本原理是碱基互补配对，分析杂交信号的强度及分布。根据应用内容不同，基因芯片可以细分为SNP基因芯片、表达谱基因芯片、微RNA（miRNA）基因芯片、DNA甲基化基因芯片、微阵列比较基因组杂交（a-CGH）基因芯片、和染色质免疫共沉淀芯片等。基因芯片具有高通量、快速等特点，可以一次大批量检测。将基因芯片与需要大样本量的全基因组关联研究（GWAS）相结合，可以发掘出更多VTE相关的基因多态性，尤其是以往所遗漏的凝血或纤溶通路以外的基因，进而为VTE发病的分子机制研究带来新的生机。

1. SNP芯片：SNP芯片技术的应用实现了低成本全基因组捕获测序，利用SNP的广泛性和遗传稳定性，可以筛查群体中病例组和对照组中的某一个等位基因频率的差异。GWAS可根据该SNP位点在基因组中的位置和连锁不平衡关系推测疾病易感基因，以实现更全面地筛选出VTE相关的SNP[12]。

与疾病直接相关的基因称为一级基因，目前已通过GWAS发现很多了基因通过参与调控一级基因来影响VTE的发生发展。在2009年，Trégouët等[13]报道了第一个和VTE相关的GWAS研究，证实FV和ABO是成人VTE的遗传易感因素。在一项随访研究中，Morange等[14]在全基因组范围内发现了新的VTE易感基因HIVEP1。Heit等[15]新发现了两个成人VTE易感基因（BLZF1、NME7）与FV和ABO基因显著关联。一项纳入了7507例VTE病例和52632例对照的GWAS研究发现了9个VTE相关位点，其中3个为新发现的位点（AFPM2、TSPAN15和SLC44A2）[16]。此外，在一项纳入212例儿童的VTE家系研究中，Rühle等[17]新发现了三个儿童VTE的易感基因（SMAP1、B3GAT2和RIMS1）。SNP芯片的廉价、快速特性使得其适用于大样本量分析，能提高GWAS从庞大基因组中检测到微小遗传信号的效能。GWAS可以通过分析SNP来挖掘VTE易感基因，为研究VTE的遗传学基础和发病机制奠定了基础。

2. 全基因表达谱芯片：基因表达谱芯片技术可以通过比较基因的表达差异来揭示疾病发生的分子机制，为基因功能研究提供思路。通过基因表达谱芯片可以进一步研究基因-基因、基因-环境因素对VTE的影响，为识别VTE的高危人群提供分子遗传学依据。

Srivastava等[18]通过全基因组表达芯片技术发现了10个基因在高海拔地区VTE患者（HA-VTE）中表达上调，差异表达的基因可能是HA-VTE发生的促进因素。同样，Lewis等[19]和Xu等[20]分别发现VTE的复发与AKT信号通路、JAK-STAT信号通路的表达上调有关，提示这些通路可能成为治疗VTE的潜在靶点。全基因组表达谱芯片可以在全基因组范围内检测与VTE相关的差异基因，是研究VTE分子机制的强有力工具，并使得预测VTE预后情况成为可能。

3. MiRNA芯片：miRNA是一种非编码RNA，通过靶向下调基因表达来执行功能。许多研究表明miRNA是血栓形成和溶解过程中的调节因子，Qin等[21]和Kessler等[22]分别发现miRNA可作为VTE急性期的生物标志物。Wang等[23]进一步研究发现miRNA能区分首发和复发的VTE，其中有14个miRNA在VTE复发患者与无复发患者之间有表达差异。在过去几年中，虽然miRNA在VTE研究中取得了一定进展，有望成为预测VTE发生和复发的生物标志物，但是各研究之间的重复性较差。因此有必要进一步对更大的样本量和不同病程的VTE患者进行研究。

4. 甲基化基因芯片：DNA甲基化是一种常见的表观基因组改变，是真核生物调节基因表达的一种过程。在甲基化芯片大批量投入使用前，已有研究表明部分编码凝血因子的基因（FⅦ[24]、FⅧ[25]）表达水平受到DNA甲基化的调节。随后一项开创性研究利用全基因组DNA甲基化分析技术，发现DNA甲基化基因cg26285502与凝血级联反应的数量性状之间有潜在关联，且这一发现在全基因组水平具有显著差异[26]。甲基化芯片技术是研究VTE复杂病因的创新手段，并为基因和环境之间作用机制提供新思考[27]。

基因芯片检测信息高通量，可检测众多变异位点，可用于临床早期诊断和批量筛选、基因表达谱测定、多态性分析等。但由于其只能检测已知位点，随着高通量测序技术的快速进展，DNA芯片在疾病基因诊断中的应用受到了一定限制。

三、以NGS为基础的分子诊断技术

凭借通量高，操作简便等特点，NGS迅速普及并快速发展、完善，为探究VTE遗传背景、协助临床诊断打开了新局面，NGS将成为VTE成因探究、个体化治疗的重要依据[28]。根据检测目标序列的不同，NGS可以分为全基因组外显子组测序（WES）、全基因组测序（WGS）、靶向测序（TS）。

（一）WES

WES是指利用序列捕获技术将全基因组外显子区域DNA捕获富集后进行测序，能发现影响蛋白质功能的遗传变异。虽然外显子约占基因组的1％，但包含85％的致病突变[29]。大量研究表明WES可以用于筛选、发现新的致病突变。Lee等[30]对64例VTE患者进行WES及分析，发现了17个新突变。此外还有一系列研究发现了VTE新的易感位点或突变[17],[31]。除了发现新的位点，WES对研究VTE发病机制也有一定启示。de la Morena-Barrio等[32]在抗凝血酶缺陷的患者中发现PMM2突变，且提示该突变会通过影响N⁃糖基化路径相关蛋白从而促进VTE发生。

（二）WGS

WES主要关注基因外显子区域，而WGS能覆盖最广泛的基因组区域，且能检测非编码区的序列。Bolze等[33]指出WES在检测编码区域的单核苷酸变异方面有优势，但在鉴定单核苷酸变异、插入和缺失突变时，WGS的结果更为稳定。虽然目前WGS成本高于WES，因此随着测序成本下降以及生物信息分析流程的成熟，从长远角度考虑，WGS更具有临床应用优势。

四项研究先后通过WGS技术发现ABO血型与VTE的相关性[13]–[15],[34]。除此之外还有一项大型研究，不仅在3855名人群中验证了先前通过GWAS发现的基因座，还发现RGS18基因突变与血栓发生风险增加有关[35]。

（三）TS

目前最常用的TS技术是基因Panel，基因Panel对已知基因突变的筛查具有明显优势，可以快速、全面地检测出目标基因，有助于对大范围人群进行特定基因位点分析。

近年，基因Panel技术在遗传性出凝血疾病中研究的数量和种类急剧增加，主要分为遗传性出血疾病[36]–[40]、遗传性血小板疾病[37]–[38],[41]–[44]、遗传性易栓症[30],[37],[45]–[48]。表1选择性地总结了基因Panel在遗传性易栓症中的应用研究，归纳了各研究的候选基因数量、纳入患者数量、诊断率。由于研究之间的患者纳入标准和候选基因数量都有差异，因此不能直接比较诊断率，但是可以看出基因Panel总体发展趋势是通过增加候选基因数量提高检出率。为了改进NGS在临床环境中的应用情况，国际血栓和止血学会和血栓形成与止血遗传学标准化委员会整理了与出血、血栓和血小板紊乱疾病相关的“诊断基因”[49]，并保持每年更新。考虑到VTE的形成涉及到多个基因位点，且随着VTE遗传背景信息的不断更新、完善，TS技术将会成为研究VTE遗传因素、筛查高危人群的不可替代的技术平台。

**表1:** 遗传性易栓症基因Panel研究报道

作者	发表年份	候选基因数量	检测人数	检出率（%）
de Visser等[46]	2013	7	201	82.1
Simeoni等[37]	2016	63	300	74.7
Lee等[30]	2017	55	64	60.9
李蕾等[48]	2019	18	246	64.6
Downes等[45]	2019	96	284	48.9
Athar等[47]	2021	18	29	75.9

（四）基因Panel与WES、WGS的比较

1. 基因Panel的局限性：首先，作为一项分子生物学技术，基因Panel的使用需要运用相关软件和具备一定的理论基础，这在一定程度上限制了基因芯片的大规模推广应用。其次，部分VTE的发生可能是由未知因素（如未知遗传调控因素）或环境因素引起的，这部分VTE很难通过基因Panel发现。再次，基因Panel只能分析已知序列的基因，不能发现新的易感基因。现阶段人们对VTE基因谱的认识还并不完全，还有很多易感基因尚未被发掘，一些突变的临床意义尚不明确。但从长远看来，基因Panel的大规模使用会积累大批量的数据结果，在这过程中会有越来越多的相关基因被发现，其作用机制也会被逐渐明确。

2. 基因Panel相对于WGS、WES的优势：WGS虽然覆盖最广泛的基因组区域，但通常平均测序深度低，发现突变的置信度可能下降，且数据分析和储存成本高。虽然与WGS相比，WES关注基因外显子区域，从而提高测序深度，成本相对较低，但是WES对非编码区上的信息了解程度有限。WES和WGS检测出的新基因与非编码区信息可以用于研究，但是其数据分析和存储更具挑战性，不适合临床应用。与这两种技术相比，基因Panel性价比更高，能快速、准确且有效地筛查VTE遗传高危因素，在早期病因诊断上有巨大发展潜力。未来，临床可根据基因检测结果，预判遗传风险的高低，为患者制定相应的预防治疗方案，预防血栓的发生或复发，降低血栓后综合征的发生率。有理由相信在后基因组时代科学研究推动下，基因Panel会被广泛推广及应用于临床。

四、展望

VTE是涉及多系统、多学科的疾病，其病因和发病机制受多基因的影响，遗传风险因素具有高度异质性[50]–[51]，为早期发现、预防VTE带来一定的难度。近年来，随着对VTE的遗传危险因素越来越了解，相关研究也在迅猛发展，但是对VTE的遗传性危险因素的检测大多处于科研阶段，尚未充分的应用到临床。当今分子诊断技术日新月异，带着VTE分子诊断向着高通量、高效、低成本发展，基因变异信息爆炸式增长，有必要建立VTE遗传信息数据库，完善遗传变异功能分析以及功能验证，阐明更多的新基因或新突变位点的临床应用价值。因此，加快推广分子诊断技术在VTE临床中的应用有望实现早期评估VTE遗传风险，从而实现筛查高危人群和早期预防血栓、靶向干预治疗。
